# Comparative assessment of the diagnostic efficacy of [^18^F]AlF-NOTA-FAPI-04 and [^18^F]FDG PET/CT imaging for detecting postoperative recurrence in gastric cancer patients: a pilot study

**DOI:** 10.3389/fonc.2024.1427649

**Published:** 2024-09-11

**Authors:** Jian Yang, Yong Wu, Yanyin Zhang, Xiang Peng, Chengzhi Jiang, Wanjing Zhou, Jiashun Dai, Aimin Xie, Hui Ye, Kai Zheng

**Affiliations:** PET/CT Center, Hunan Cancer Hospital/The Affiliated Cancer Hospital Of Xiangya School Of Medicine, Central South University, Changsha, China

**Keywords:** [^18^F]FDG, [^18^F]AlF-NOTA-FAPI-04, PET/CT, gastric cancer, recurrence

## Abstract

**Purpose:**

This study aimed to compare the efficacy of [^18^F]AlF-NOTA-FAPI-04 PET/CT with that of [^18^F]FDG PET/CT for detecting postoperative recurrence in patients with gastric cancer.

**Methods:**

This single-center retrospective clinical study was performed at Hunan Cancer Hospital between December 2020 and June 2022. The participants underwent both [^18^F]AlF-NOTA-FAPI-04 and [^18^F]FDG within 14 days. Histopathologic examination, morphological imaging, and/or follow-up imaging were used as a reference for the final diagnosis. We recorded the sensitivity, specificity, positive predictive value (PPV), negative predictive value (NPV) and accuracy of [^18^F]AlF-NOTA-FAPI-04 and [^18^F]FDG PET/CT for detecting local recurrence, lymph node metastasis and distant metastasis. The SUVmax and background ratio (TBR) of local recurrence and metastases between [^18^F]FDG and [^18^F]AlF-NOTA-FAPI-04 PET/CT were compared using paired-sample t tests.

**Results:**

Forty-seven patients (27 males, aged 25–68 years) with gastric cancer after curative resection (27 with adenocarcinoma, 17 with signet ring cell carcinoma and 4 with mucinous adenocarcinoma) were included in the study. [^18^F]AlF-NOTA-FAPI-04 accumulation was significantly greater than that of [^18^F]FDG in terms of local recurrence (SUVmax, 11.65 vs 3.48, p< 0.0001; TBR, 12.93 vs 2.94, p< 0.0001), lymph node metastasis (SUVmax, 13.45 vs 3.05, p=0.003875; TBR, 12.43 vs 2.21, p=0.001661), and distant metastasis (SUVmax, 11.89 vs 2.96, p < 0.0001; TBR, 13.32 vs 2.32, p< 0.0001). Despite no statistical comparison was made with [^18^F]FDG, [^18^F]AlF-NOTA-FAPI-04 imaging exhibited high levels of sensitivity, specificity, PPV, NPV, and accuracy for detecting postoperative local recurrence, lymph node metastasis, and distant metastasis in patients with gastric cancer.

**Conclusion:**

[^18^F]AlF-NOTA-FAPI-04 has demonstrated potential for more accurate tumor re-evaluation in GC, thus enhancing treatment decision-making.

## Introduction

Gastric cancer (GC) ranks as the fifth most common cancer in China in 2022, accounting for 7.4% of the total new cancer cases, and ranks third in terms of estimated numbers of cancer deaths and cancer mortality rates (1). Although surgical resection remains the primary curative approach for GC, long-term survival rates remain unsatisfactory. The conventional diagnosis of tumor recurrence and metastasis is paramount for effective GC patient management. However, traditional imaging techniques, including gastroscopy, X-ray computed tomography (CT), magnetic resonance imaging (MRI) and endoscopic ultrasonography (EUS), have inherent limitations, particularly in detecting occult lymph nodes and peritoneal carcinomatosis (2, 3).

[^18^F]Fluorine fluorodeoxyglucose positron emission tomography/computed tomography (PET/CT) has been employed for diagnosing various cancers, including GC (4, 5). Nevertheless, certain histological subtypes, such as signet ring cell adenocarcinomas (SRCC) and mucinous adenocarcinoma (MAC), may exhibit limited avidity for [^18^F]FDG (6, 7). Additionally, physiological [^18^F]FDG uptake in the gastrointestinal tract can interfere with accurate lesion detection (8). These limitations may impede the detection of local recurrence or metastatic lesions of GC using [^18^F]FDG PET/CT, thereby complicating the confirmation of recurrence and potentially delaying treatment (9). Consequently, there is a pressing need for the development of an effective PET tracer that overcomes the limitations of [^18^F]FDG.

Fibroblast-activated protein (FAP), typically overexpressed in cancer-associated fibroblasts within the tumor stroma, represents a promising target for tumor imaging (10). Radionuclide-labelled fibroblast-activated protein inhibitors, such as [^68^Ga]fibroblast-activated protein inhibitor ([^68^Ga]Ga-FAPI), have emerged as PET tracers targeting FAP, demonstrating superiority over [^18^F]FDG in imaging various cancers (11, 16, 17). [^68^Ga]Ga-FAPI has been validated in multiple studies for initial staging, restaging, and prognostic evaluation of GC, as well as for predicting pathological response to neoadjuvant chemotherapy (18, 19). Compared to the widely used [^68^Ga]labeled ligands, [^18^F]fluorine offers several significant advantages, including increased examination capacity due to enhanced production capabilities, as well as superior image quality (13, 15). Recently, [^18^F]labelled tracers targeting FAPI, including [^18^F]AlF-1,4,7-triazacyclononane-l,4,7-triacetic acid-fibroblast activation protein inhibitor-04 ([^18^F]AlF-NOTA-FAPI-04), have been proposed for clinical use in various cancers, including GC (12–14). However, the specific role of [^18^F]AlF-NOTA-FAPI-04 PET/CT in detecting postoperative local recurrence and metastasis in GC patients, and whether it offers advantages over [^18^F]FDG imaging, has not been reported. This study aimed to evaluate the utility of [^18^F]AlF-NOTA-FAPI-04 PET/CT in patients suspected of having GC recurrence.

## Materials and methods

### Patients

We retrospectively analysed 47 patients who underwent radical gastrectomy and were suspected of having GC recurrence at our institution between December 2020 and August 2022. All patients underwent [^18^F]FDG and [^18^F]AlF-NOTA-FAPI-04 PET/CT imaging within 14 days for recurrence diagnosis. The indications for PET/CT scans were categorized into three groups: Group 1 (n=25) comprised patients with clinical manifestations such as weight loss, abdominal distention, or pain; Group 2 (n=13) included those suspected of having recurrence due to elevated tumor markers without conclusive prior imaging or clinical symptoms; and Group 3 (n=9) encompassed patients suspected of having recurrence based on other imaging modalities, such as CT or MRI. Patients who had undergone adjuvant chemotherapy or radiotherapy within 4 weeks prior to the PET/CT scan were excluded. The final recurrence diagnosis was confirmed through histopathological examination following surgery, laparotomy, biopsy, or clinical follow-up lasting at least 6 months.

### Preparation of [^18^F]FDG and [^18^F]AlF-NOTA-FAPI-04

Using a GE MINItrace cyclotron system (GE, USA), the F-18 tracer was generated *in situ* through bombardment of [^18^O]-H2O with protons at 9.8 MeV. Strict adherence to the established protocol was observed during the synthesis of [^18^F]FDG, leveraging the coincidental [^18^F]FDG synthesis module (AIO; TRSIS, China). The precursor FAPI-04 was obtained from PET (Beijing) Science and Technology Co., Ltd. The labelling of [^18^F]AlF-NOTA-FAPI-04 followed the method described by Jiang et al. (14). Both [^18^F]AlF-NOTA-FAPI-04 and [^18^F]FDG exhibited radiochemical purities exceeding 95%. Prior to its application, the sterile final product met all institutional standards.

### PET/CT imaging

Paired [^18^F]FDG and [^18^F]AlF-NOTA-FAPI-04 PET/CT scans were obtained within a 14-day period. Prior to the [^18^F]FDG PET/CT scan, patients were instructed to fast for at least 6 hours and consume 500 mL of water to aid renal clearance of both tracers. Peripheral blood glucose levels were assayed to ensure normoglycaemia before imaging. The intravenous doses of [^18^F]FDG and [^18^F]AlF-NOTA-FAPI-04 were customized based on the patient’s body weight and were administered at a concentration of 3.7 MBq (0.1 mCi)/kg for both tracers.

One hour after intravenous administration, data acquisition was performed on a Discovery MI hybrid PET/CT scanner (GE Healthcare, Milwaukee, WI, USA). For [^18^F]AlF-NOTA-FAPI-04 and [^18^F]FDG imaging, the CT scan encompassed the cranial vertex to the upper thighs, utilizing a tube voltage of 110 kV, a tube current of 120 mA, and a slice thickness of 3.75 mm.

Subsequently, a PET scan in 3D acquisition mode was conducted, encompassing 5-6 bed positions with a duration of 2 minutes per position. The acquired data were transferred to an Advantage Workstation (version AW 4.7, GE Healthcare, Milwaukee, WI, USA) and reconstructed using the ordered subset expectation maximization (OSEM) algorithm, with two iterations and 21 subsets. The CT data were used for attenuation correction, and the resulting images were coregistered for analysis.

### Imaging analysis

Two experienced nuclear medicine physicians, each with over five years of specialization in nuclear oncology, independently evaluated all [^18^F]AlF-NOTA-FAPI-04 and [^18^F]FDG PET/CT scans. Any discrepancies in their assessments were resolved through discussion to reach a consensus. The interpretation process encompassed both visual and semiquantitative analyses. Positive lesions were recognized as regions exhibiting increased radioactivity, excluding physiological uptake, relative to the background. To quantify the uptake levels of these lesions, we employed the maximum standardized uptake value (SUVmax) for both the [^18^F]AlF-NOTA-FAPI-04 and [^18^F]FDG PET/CT scans. Conversely, the quantification of healthy organs was based on the SUVmean. SUV values were determined by positioning volumes of interest (VOIs) on metastatic lesions observed on the scans. Circular regions of interest (ROIs) were strategically placed on axial slices surrounding avid-lesion areas and automatically integrated into a 3D VOI using the AW system (GE, USA). The SUVmax and tumor-to-background ratio (TBR) of patients with locoregional recurrence, lymph node metastasis, peritoneal carcinomatosis, and bone metastasis were recorded for both the [^18^F]AlF-NOTA-FAPI-04 and [^18^F]FDG PET/CT images. In cases where multiple positive lesions were present, the lesions exhibiting the highest SUVmax and TBR values were prioritized for recording. Tracer uptake was qualitatively deemed positive when focal uptake exceeded normal background activity. For statistical analysis, lesions were classified as follows: true-positive lesions were those observed on PET/CT images and histopathologically or clinically confirmed as tumor tissue; false-positive lesions appeared on PET/CT images but were histopathologically or clinically determined to be nontumorous; true-negative lesions were undetected on PET/CT images and confirmed as negative through histopathological examination or clinical follow-up; and false-negative lesions were those not identified during image analysis but later histopathologically or clinically confirmed as malignant.

### Statistical analysis

The primary objective of this study was to document the sensitivity, specificity, positive predictive value (PPV), negative predictive value (NPV), and accuracy in detecting local recurrence, lymph node metastasis, and distant metastases using [^18^F]AlF-NOTA-FAPI-04 and [^18^F]FDG PET/CT, with histopathology serving as the gold standard. The secondary aim was to compare tumor SUV_max_ and TBR. For the statistical analysis, we used SPSS (version 25.0; SPSS Inc., Chicago, IL, USA). Categorical variables are presented as frequencies and percentages, while continuous variables are expressed as the means ± standard deviations (SDs). To compare the mean SUVmax among different categorical groups and the TBRs between [^18^F]AlF-NOTA-FAPI-04 and [^18^F]FDG in the primary tumor, we utilized paired sample t tests. A p-value less than 0.05 was considered to indicate statistical significance.

## Results

### Patient characteristics

The clinical characteristics of the 47 patients included 25 (53.19%) males and 22 (46.81%) females with a mean age of 52.26 ± 10.2 years (ranging from 25 to 68 years). Of these patients, 26 were diagnosed with adenocarcinoma, 17 with signet ring cell carcinoma, and 4 with mucinous adenocarcinoma. All 47 patients underwent radical surgery, and 34 patients received initial standard adjuvant therapy, including the SOX, FOLFOX, or XELOX regimens, following gastrectomy. Notably, 3 patients who received adjuvant chemotherapy also underwent adjuvant radiotherapy. Conversely, the remaining 13 patients did not receive adjuvant chemotherapy due to their early-stage disease. Histologically, 13 patients exhibited moderately to poorly differentiated tumors, while 34 patients had poorly differentiated tumors, encompassing 13 adenocarcinomas, 17 SRCC, and 4 MAC. The comprehensive patient characteristics are detailed in **Table 1**.

**Table 1:** Patient characteristics (n = 47).

Characteristics	Value
Age (year)	52.26 ± 10.2 (25, 68)
Sex	
F	22
M	25
Histology	
Adenocarcinoma	26
Signet ring cell carcinoma	17
Mucinous adenocarcinoma	4
Degree of differentiation	
Moderately-poorly	13
Poorly	34
PET/CT detection	
[^18^F]FDG	
Positive	27 (57.4%)
Negative	20 (42.6%)
[^18^F]AlF-NOTA-FAPI-04	
Positive	38 (80.8%)
Negative	9 (19.1%)
Post-scan treatment	
Surgery	13
Surgery+chemotherapy	31
Surgery+chemotherapy+radiotherapy	3

### Diagnostic performance of [^18^F]FDG and [^18^F]AlF-NOTA-FAPI-04 PET/CT for locoregional recurrence

**Figure 1:**
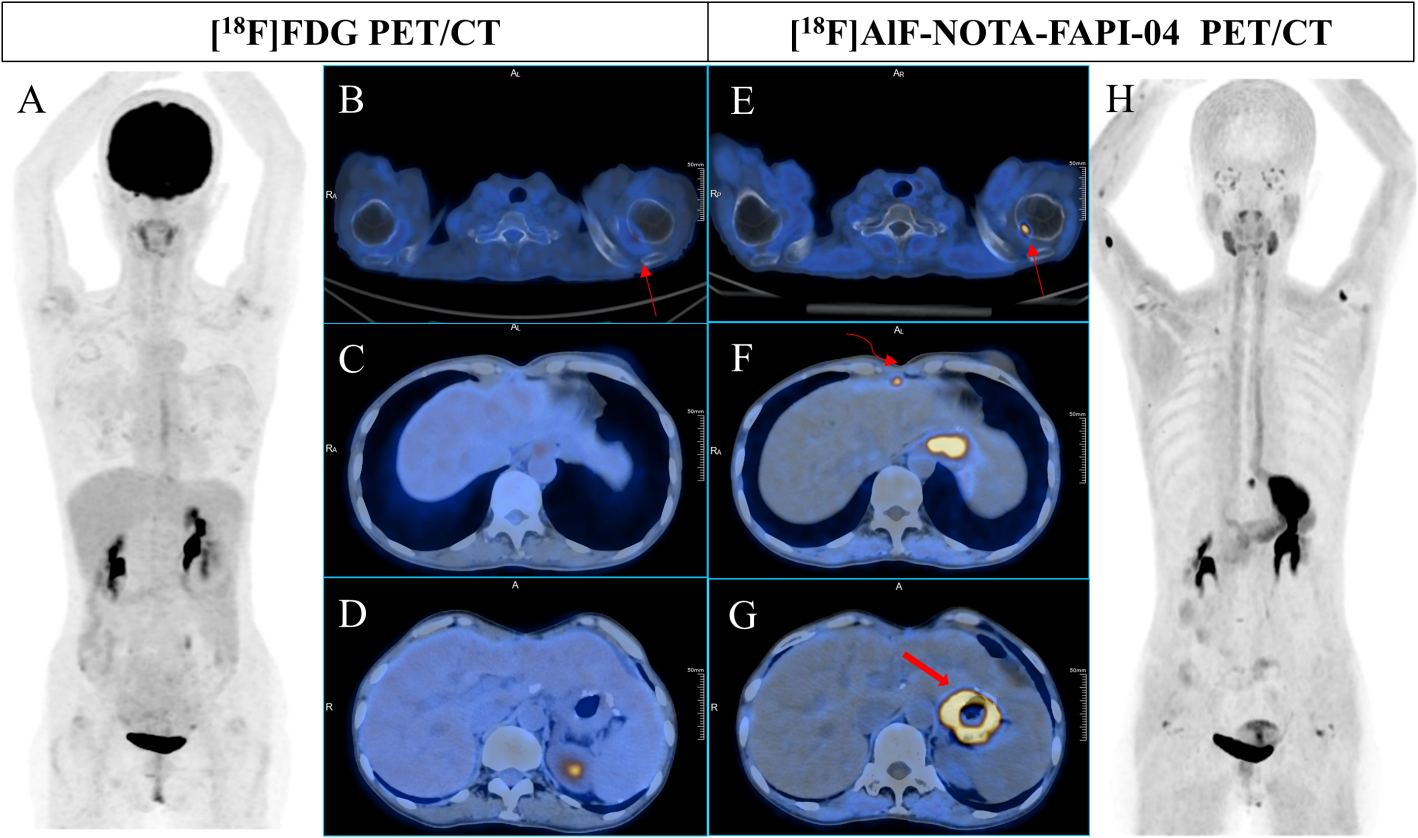
A 51-year-old woman underwent radical resection for poorly differentiated gastric adenocarcinoma thirteen months prior, followed by 4 cycles of FOLFIRI + cetuximab and 4 cycles of XELOX + cetuximab postoperative therapy. During routine follow-up, gastric endoscopy confirmed anastomotic recurrence. Subsequent [^18^F]FDG PET/CT **(A-D)** scans showed no abnormal uptake in the entire body. However, [^18^F]AlF-NOTA-FAPI-04 PET/CT **(E-H)** revealed increased tracer uptake in the right internal mammary lymph node (**F**, curved arrow, SUVmax 8.5) and thickening at the site of anastomosis (**F, G**, bold arrow, SUVmax 15.8). Additionally, [^18^F]AlF-NOTA-FAPI-04 PET/CT (**E**, thin arrow, SUVmax 10.9) indicated significantly high uptake at the inner edge of the right humeral head, while [18F]FDG PET/CT showed only mild uptake (**B**, curved arrow, SUVmax 3.4), consistent with the degenerative bone disease observed in previous contrast-enhanced CT images.

In diagnosing local recurrence, [^18^F]FDG PET/CT exhibited a sensitivity of 46.66% (7/15), a specificity of 96.87% (31/32), a PPV of 87.5% (7/8), an NPV of 79.48% (31/39), and an accuracy of 80.85% (38/47). [^18^F]AlF-NOTA-FAPI-04 PET/CT exhibited a sensitivity of 100% (15/15), a specificity of 93.75% (30/32), a PPV of 88.23% (15/17), an NPV of 100% (30/30), and an overall accuracy of 95.74% (45/47), as detailed in **Table 2**. Among the cohort of 47 GC patients, one patient with SRCC exhibited positive uptake of both [^18^F]FDG and [^18^F]AlF-NOTA-FAPI-04 in chronic active inflammation of the anastomotic stoma mucosa. Another patient with SRCC had negative [^18^F]FDG uptake for postoperative residual gastritis, whereas [^18^F]AlF-NOTA-FAPI-04 had positive uptake. For the remaining 45 patients, [^18^F]AlF-NOTA-FAPI-04 imaging revealed 30 true negatives and 15 true positives, whereas [^18^F]FDG demonstrated 8 false negatives, 31 true negatives, and 7 true positives (**Figure 1**). Notably, among the 8 false-negative patients who underwent [^18^F]FDG recurrence detection, 5 were diagnosed with SRCC, 1 with MAC, and 2 with poorly differentiated adenocarcinoma. During follow-up, 15 patients (31.91%) were confirmed to have local recurrence through gastroscopy or surgical biopsy. Notably, the uptake of [^18^F]AlF-NOTA-FAPI-04 was significantly greater in patients with local recurrence, with an SUVmax of 11.65 ± 4.44 and a TBR of 12.93 ± 5.37, than in those with [^18^F]FDG (3.48 ± 1.71 and 2.94 ± 1.47, respectively). All p values were less than 0.0001 (**Table 3**).

**Table 2:** Diagnostic performance of [^18^F]FDG and [^18^F]AlF-NOTA-FAPI-04 PET/CT.

	True positive (n)	True negative (n)	False positive (n)	False negative (n)	Sensitivity (%)	Specificity (%)	Positive predictive value (%)	Negative predictive value (%)	Accuracy (%)
Local recurrence									
[^18^F]FDG	7	31	1	8	46.66	96.87	87.5	79.48	80.85
[^18^F]AlF-NOTA-FAPI-04	15	30	2	0	100	93.75	88.23	100	95.74
Lymph node metastasis									
[^18^F]FDG	3	35	1	8	27.27	97.22	75	81.39	86.36
[^18^F]AlF-NOTA-FAPI-04	11	36	0	0	100	100	100	100	100
Distant metastasis									
[^18^F]FDG	16	15	1	15	51.61	93.75	94.11	53.33	65.95
[^18^F]AlF-NOTA-FAPI-04	30	16	0	1	96.77	94.11	100	94.11	97.87

**Table 3:** Detection presentation in [^18^F]FDG and [^18^F]AlF-NOTA-FAPI-04 PET/CT imaging for patients suspicious for gastric cancer recurrence (n = 47).

Detection	No. Of patients	SUVmax			TBR		
		[^18^F]FDG	[^18^F]AlF-NOTA-FAPI-04	p	[^18^F]FDG	[^18^F]AlF-NOTA-FAPI-04	p
Local recurrence	15	3.48 ± 1.71	11.65 ± 4.44	<0.0001	2.94 ± 1.47	12.9 ± 5.37	<0.0001^*^
Lymph node	12	3.05 ± 2.56	11.45 ± 7.59	0.003875	2.21 ± 1.78	12.43 ± 8.46	0.001661^*^
Distant metastasis	31	2.96 ± 2.21	11.89 ± 5.07	0.003875	2.32 ± 1.84	13.32 ± 6.01	0.001661^*^
Peritoneal	25	2.5 ± 1.43	11.93 ± 4.8	<0.0001	1.91 ± 0.98	13.14 ± 5.26	<0.0001^*^
Ovary	7	3.83 ± 3.47	9.24 ± 3.13	0.012	2.84 ± 3.22	9.96 ± 4.77	0.009^*^
Bone	3	5.9 ± 3.46	12.4 ± 8.45	–	5.9 ± 3.46	24.17 ± 13.16	–

TBR, tumor-to-background ratio.

### Diagnostic performance of [^18^F]FDG and [^18^F]AlF-NOTA-FAPI-04 PET/CT in nodal metastasis

**Figure 2:**
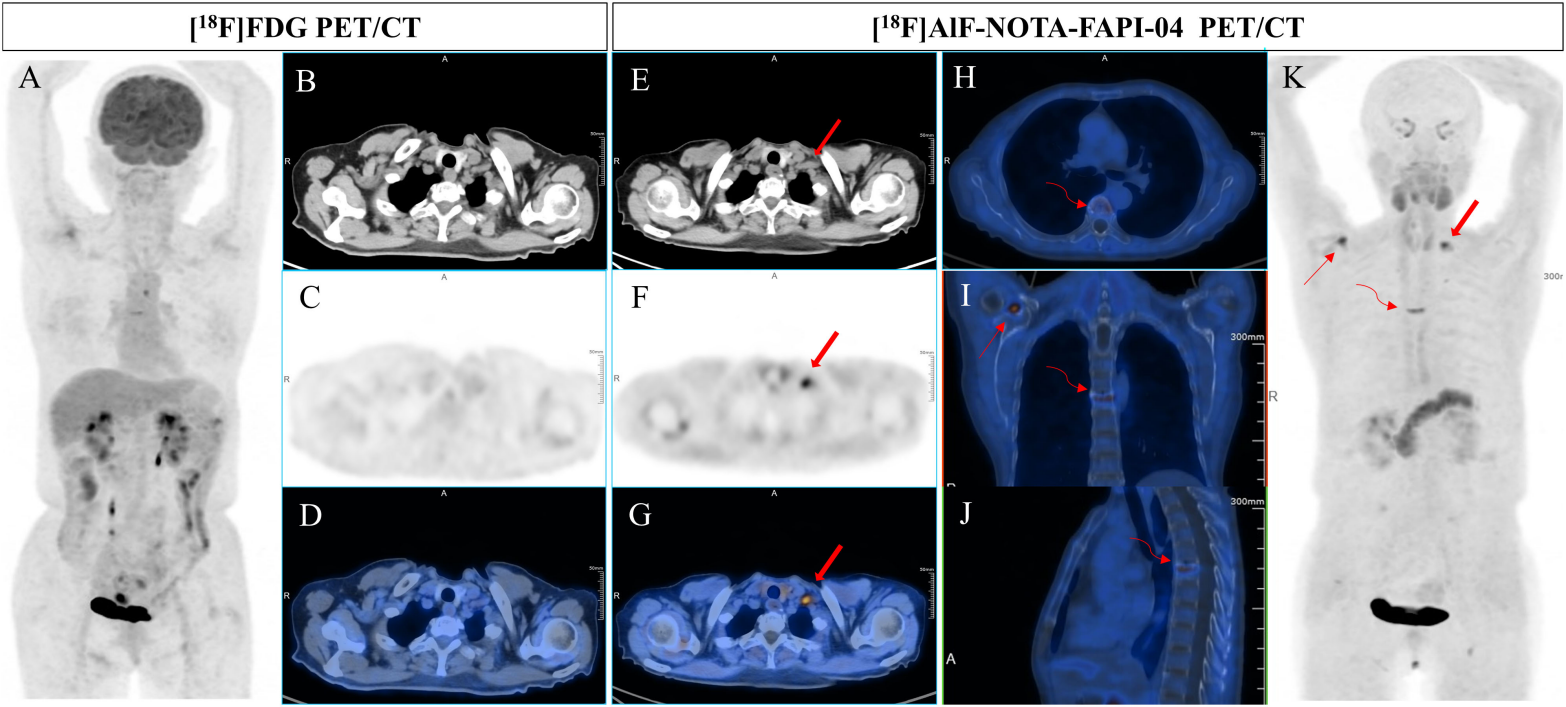
A 61-year-old woman underwent gastric radical resection for a cancer of nine months old and received 6 cycles of SOX therapy. Elevated levels of CEA, CA199, and CA50 (9.5 ng/mL, 98.35 U/mL, and 48.4 IU/mL, respectively) were detected during routine follow-up. To detect potential metastases, she underwent [^18^F]FDG PET/CT **(A-D)** examination, which revealed no abnormal uptake in the entire body. However, [^18^F]AlF-NOTA-FAPI-04 PET/CT **(E-H)** showed increased tracer uptake in the left supraclavicular lymph node (bold arrow, the SUVmax of 7.9). Additionally, subsequent contrast-enhanced CT demonstrated significant enlargement of the lesion. In addition, [18F]AlF-NOTA-FAPI-04 imaging revealed abnormal uptake in the degenerative bone disease of the sixth thoracic vertebra (**H-K**, curved arrow) and in the right shoulder joint due to arthritis (**I, K**, thin arrow), with SUVmax of 6.5 and 7.2, respectively.

[^18^F]FDG PET/CT demonstrated a sensitivity of 27.27% (3/11), specificity of 97.22% (25/36), PPV of 75% (3/4), NPV of 81.39% (35/43), and accuracy of 86.36% (38/47) in diagnosing lymph node metastasis. Conversely, [^18^F]AlF-NOTA-FAPI-04 PET/CT exhibited a sensitivity of 100% (11/11), specificity of 100% (36/36), PPV of 100% (36/36), NPV of 100% (11/11), and perfect accuracy (47/47) (**Table 2**). Among the 47 patients enrolled, 3 had concordant true positive results according to both modalities, while 35 patients consistently had true negative results. Eight patients had true positive results only for [^18^F]AlF-NOTA-FAPI-04, while [^18^F]FDG yielded false negative results (**Figure 2**). Additionally, [^18^F]FDG generated one false positive, whereas [^18^F]AlF-NOTA-FAPI-04 accurately indicated a true negative. During follow-up, 11 patients were diagnosed with metastasis, and [^18^F]AlF-NOTA-FAPI-04 PET/CT revealed significantly greater tracer uptake, with a greater TBR than [^18^F]FDG PET/CT (SUVmax, 11.45 ± 7.59 vs 3.05 ± 2.56, p =0.003875; TBR, 12.43± 8.46 vs 2.21 ± 1.78, p =0.001661) (**Table 3**).

### Diagnostic performance of [^18^F]FDG and [^18^F]AlF-NOTA-FAPI-04 PET/CT in distant metastasis

[^18^F]FDG PET/CT exhibited a sensitivity of 51.61% (16/31), a specificity of 93.75% (15/16), a PPV of 94.11% (16/17), an NPV of 53.33% (16/30), and an accuracy of 65.95% (31/47). Conversely, [^18^F]AlF-NOTA-FAPI-04 PET/CT demonstrated a sensitivity of 96.77% (30/31), a specificity of 94.11% (16/17), a PPV of 100% (30/30), an NPV of 94.11% (16/17), and an accuracy of 97.87% (46/47) (**Table 2**).

**Figure 3:**
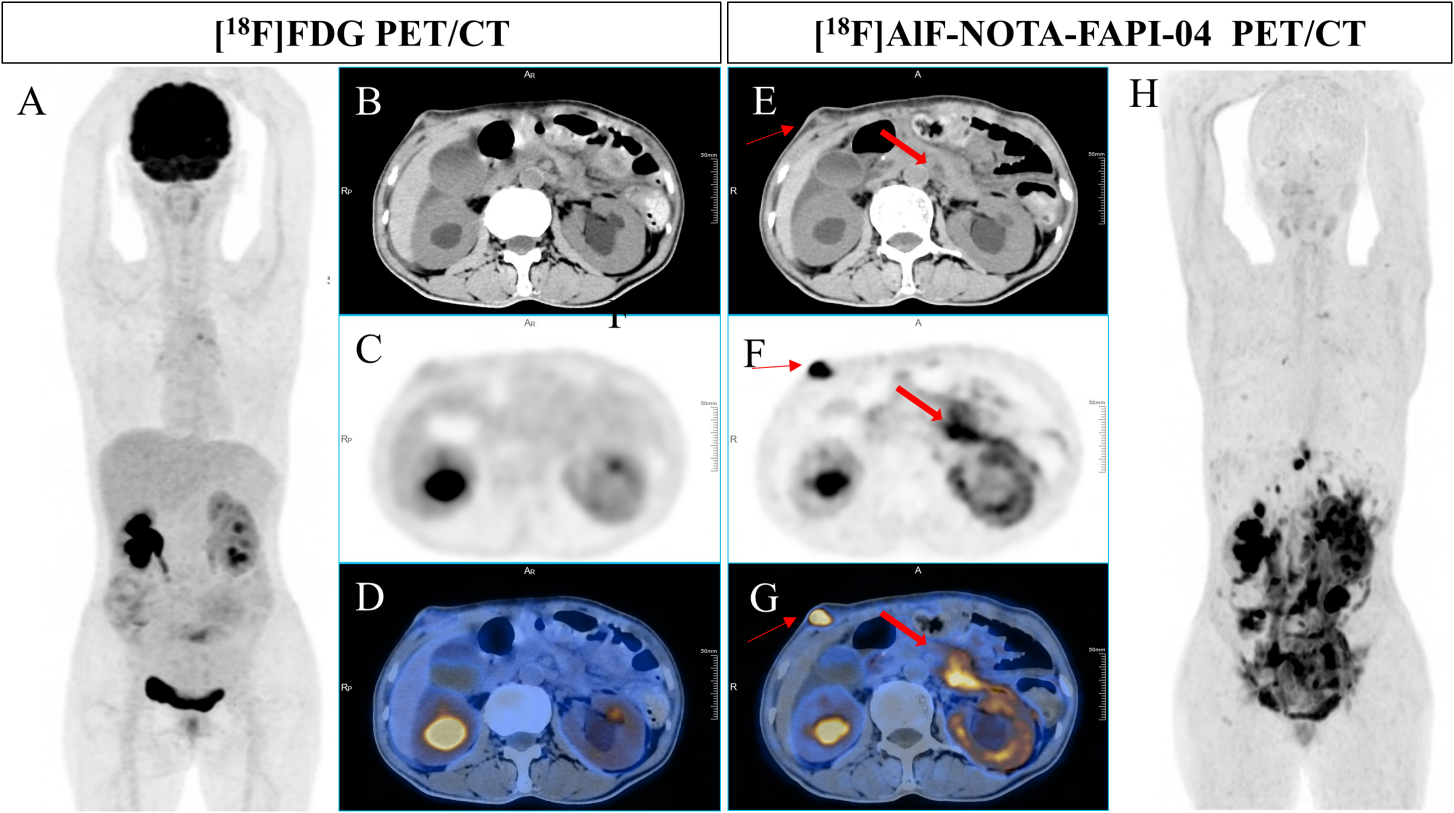
A 49-year-old man underwent radical resection for poorly differentiated adenocarcinoma of the stomach twelve months prior. Subsequent routine CT scans revealed suspected peritoneal carcinomatosis. [^18^F]FDG PET/CT imaging **(A-D)** demonstrated no abnormal uptake throughout the whole body. However, [^18^F]AlF-NOTA-FAPI-04 PET/CT **(E-H)** revealed increased tracer uptake in the thickened retroperitoneal soft tissue and a mass in the right abdominal wall (bold arrow, SUVmax of 14.5; thin arrow, SUVmax of 21.9). Pathological examination of the biopsy sample from the mass in the right abdominal wall indicated a few atypical round cells, suggesting malignancy.

Sixteen patients showed concordant true positive results in both modalities, while another 16 patients consistently exhibited true negative results. Additionally, 14 patients had true positive results only with [^18^F]AlF-NOTA-FAPI-04, whereas [^18^F]FDG showed false negative results (**Figure 3**). Notably, one patient exhibited false negatives in both modalities despite adenocarcinoma detection via ascitic fluid cytology. Overall, 31 patients were diagnosed with distant metastasis. Notably, both the SUVmax and TBR of [^18^F]AlF-NOTA-FAPI-04 were significantly greater than those of [^18^F]FDG (SUVmax: 11.89 ± 5.07 vs 2.96 ± 2.21, p=0.003875; TBR: 13.32 ± 6.01 vs 2.32 ± 1.84, p=0.001661) (**Table 3**).

Among 25 patients confirmed to have peritoneal carcinomatosis, [^18^F]AlF-NOTA-FAPI-04 PET/CT showed a significantly greater SUVmax (11.93 ± 4.8) and TBR (13.14 ± 5.26) than did [^18^F]FDG (SUVmax: 2.5 ± 1.43; TBR: 1.91 ± 0.98), with both differences being statistically significant (p<0.0001 for both). Thirteen patients exhibited true positive results on both imaging modalities, encompassing six adenocarcinomas, six SRCCs, and one MAC. [^18^F]FDG imaging incorrectly yielded false-negative results in twelve patients (seven patients with adenocarcinomas and five patients with SRCC), whereas [^18^F]AlF-NOTA-FAPI-04 imaging accurately revealed true-positive results. In one SRCC patient, abnormal FDG uptake was observed in the omentum, whereas [^18^F]AlF-NOTA-FAPI-04 imaging did not show any abnormal tracer accumulation. Subsequent clinical follow-up did not indicate metastasis.

Seven patients were diagnosed with ovarian metastasis. The SUVmax and TBR for [^18^F]FDG and [^18^F]AlF-NOTA-FAPI-04 PET/CT images were 3.83 ± 3.47 and 9.24 ± 3.13 and 2.84 ± 3.22 and 9.96 ± 4.77, respectively, exhibiting statistically significant differences (p=0.012 and p=0.009, respectively). Among these, five patients showed concordant true-positive results, including three with adenocarcinomas, one with SRCC, and one with MAC. Additionally, two adenocarcinomas were falsely negative on [^18^F]FDG but were positively detected on [^18^F]AlF-NOTA-FAPI-04.

Finally, three bone metastasis patients were diagnosed, with SUVmax and TBR values of 5.9 ± 3.46, 12.4 ± 8.45 and 5.9 ± 3.46, 24.17 ± 13.16 for [^18^F]FDG and [^18^F]AlF-NOTA-FAPI-04, respectively (**Table 3**). Notably, both modalities confirmed SRCC in two patients, while [^18^F]FDG incorrectly indicated a false-negative result for one patient with adenocarcinoma, which contradicted [^18^F]AlF-NOTA-FAPI-04’s true-positive finding.

### Physiological uptake in healthy organs and false-positive uptake in benign lesions

Physiological uptake was observed in the bilateral submandibular glands, thyroid, biliary tract, pancreas, and bladder in most patients. Some patients also presented with incidental benign bone lesions, including degenerative bone disease and arthritis (**Figures 1**-**3**).

## Discussion

Accurate postoperative follow-up and early recurrence detection are crucial for the effective management of GC patients after radical gastrectomy. While [^68^Ga]Ga-FAPI has attracted considerable research interest, [^18^F]AlF-NOTA-FAPI-04 remains a promising candidate and potentially serves as an alternative to [^18^F]FDG (12). [^18^F]AlF-NOTA-FAPI-04 imaging demonstrated exceptional sensitivity, specificity, PPV, NPV, and accuracy in detecting postoperative local recurrence, lymph node metastasis, and distant metastasis of GC. Therefore, the use of [^18^F]AlF-NOTA-FAPI-04 PET/CT imaging has the potential to significantly improve the postoperative clinical management of GC patients.

After rigorous analysis, [^18^F]AlF-NOTA-FAPI-04 imaging was proven to exhibit excellent diagnostic performance in the detection of local recurrence in GC patients. This finding suggests its promising application in the clinical management of patients post-GC surgery. Notably, among the eight patients with false-negative [^18^F]FDG results, five were diagnosed with SRCC, and one was diagnosed with MAC, reflecting the limitations of the tracer in detecting these specific pathological subtypes, as reported in previous literature (20–22). This reduced diagnostic sensitivity of [^18^F]FDG is attributed to the low glucose transporter 1 (GLUT-1) expression observed in the SRCC and MAC (23, 24). Additionally, [^18^F]FDG uptake in the gastrointestinal tract exhibits considerable variability (8). In contrast, [^18^F]AlF-NOTA-FAPI-04 benefits from increased FAP expression in gastrointestinal and metastatic tumor matrices, rapid renal clearance, reduced physiological uptake in normal organs, and a higher TBR, making it advantageous for abdominal and pelvic imaging (16, 25). However, it is noteworthy that [^18^F]AlF-NOTA-FAPI-04 imaging also demonstrated anomalous uptake associated with chronic active inflammation of the anastomotic stoma mucosa in one patient and with uptake related to postoperative residual gastritis in another patient, emphasizing the need for cautious interpretation, as abnormal uptake does not conclusively indicate recurrence (26, 27).

Accurate staging of lymph nodes holds paramount importance in guiding the management and prognosis of GC patients. In this study, [^18^F]AlF-NOTA-FAPI-04 PET/CT demonstrated superior efficacy over [^18^F]FDG PET/CT in detecting metastatic lymph nodes, primarily attributed to the limited uptake of [^18^F]FDG in numerous lymph nodes. The enhanced detection capability of [^18^F]AlF-NOTA-FAPI-04 PET/CT significantly contributes to precise lymph node staging and aids clinicians in devising optimal postoperative treatment strategies. Notably, [^18^F]AlF-NOTA-FAPI-04 imaging accurately visualized positive lymph node pathology in 11 patients and negative findings in 36 patients. Conversely, [^18^F]FDG imaging yielded 8 false-negative results and 1 false-positive result, the latter attributed to aberrant uptake in reactive mesenteric lymph node hyperplasia. Although inflammatory lymph nodes have been reported to exhibit [^18^F]AlF-NOTA-FAPI-04 uptake in previous studies, this observation was not observed in our study, possibly due to the limited sample size or variations in tracer performance between [^18^F]AlF-NOTA-FAPI-04 and [^68^Ga]Ga-FAPI (28, 29). Further confirmation through extensive research with expanded sample sizes is imperative (26, 27).

In detecting distant metastases, [^18^F]AlF-NOTA-FAPI-04 imaging accurately identified lesions in 14 patients; however, [^18^F]FDG imaging resulted in false-negative findings. Notably, one adenocarcinoma patient exhibited abnormal [^18^F]FDG uptake in the omentum, yet [^18^F]AlF-NOTA-FAPI-04 imaging revealed no abnormal activity. Subsequent clinical and radiological follow-ups did not indicate disease progression. Additionally, while both imaging modalities yielded false negatives in one SRCC patient, exfoliative cytological examination of the ascites fluid revealed adenocarcinoma cells. These findings underscore the distinct advantage of [^18^F]AlF-NOTA-FAPI-04 over [^18^F]FDG in postoperative imaging of distant metastases in gastric cancer, particularly for SRCC. In terms of peritoneal metastasis detection, [^18^F]FDG generated 12 false negatives and 1 false positive, emphasizing the superior detection capability of [^18^F]AlF-NOTA-FAPI-04 for peritoneal carcinomatosis among GC patients, which is consistent with recent scientific findings (22). Furthermore, in a cohort of 7 patients with confirmed ovarian metastasis, [^18^F]FDG imaging failed to detect two adenocarcinomas, further highlighting the superiority of [^18^F]AlF-NOTA-FAPI-04 in identifying ovarian involvement. This discrepancy may stem from [^18^F]FDG’s tendency for physiological uptake by the ovaries, whereas [^18^F]AlF-NOTA-FAPI-04 exhibits a heightened sensitivity to metastasis and reduced false positive rates due to physiological processes (30, 31). Regarding bone metastases, [^18^F]FDG imaging revealed no cases of adenocarcinoma, but subsequent radiological follow-up revealed metastatic deposits in the right clavicle, multiple right ribs, and the right iliac bone. Prior research has indicated that FAPI imaging has high sensitivity for detecting bone lesions; however, benign conditions can mimic pathological uptake, necessitating cautious interpretation when utilizing [^18^F]AlF-NOTA-FAPI-04 imaging for identifying bone metastases. Furthermore, multiple imaging techniques are recommended for comprehensive evaluation (32–34). Notably, within this study’s scope, there were no patients who presented with liver or lung metastases, which may be partially attributed to specific biases observed during patient selection during the recruitment period.

In our study, [^18^F]AlF-NOTA-FAPI-04 demonstrated promising outcomes in detecting local GC recurrence, nodal metastasis, and distant metastasis. Additionally, prior research has highlighted the significant advantages of [^18^F]AlF-NOTA-FAPI-04 compared to [^18^F]FDG, including enhanced yield, superior imaging clarity, improved tumor-to-background contrast, independence from blood glucose concentrations, and the capability for rapid image acquisition (13, 15, 35).

Our study is subject to several limitations. First, the sample size was relatively small, encompassing only 47 participants. Second, technical and ethical considerations precluded biopsies for all lymph nodes and distant metastases. Hence, histopathological verification of the majority of positive lesions was not possible, necessitating the use of morphological and/or follow-up imaging as surrogate reference standards. Third, the absence of histopathological data precluded lesion-specific statistical analysis. Finally, the ability to evaluate potential false-negative lesions is incomplete due to the primary reliance on noninvasive imaging for tumor staging.

## Conclusion

[^18^F]AlF-NOTA-FAPI-04 PET/CT has exhibited promising potential in facilitating more precise tumor reassessment in gastric cancer, thereby improving the process of therapeutic decision-making.

## Data Availability

The original contributions presented in the study are included in the article/supplementary material, further inquiries can be directed to the corresponding author/s.
